# Genome-wide identification and expression profile of the MADS-box gene family in *Erigeron breviscapus*

**DOI:** 10.1371/journal.pone.0226599

**Published:** 2019-12-20

**Authors:** Wen Tang, Yayi Tu, Xiaojie Cheng, Lili Zhang, Hengling Meng, Xin Zhao, Wei Zhang, Bin He

**Affiliations:** 1 Jiangxi Key Laboratory of Bioprocess Engineering and Co-Innovation Center for In-vitro Diagnostic Reagents and Devices of Jiangxi Province, College of Life Sciences, Jiangxi Science & Technology Normal University, Nanchang, China; 2 Key Laboratory of Bio-resources and Eco-environment, Ministry of Education, Sichuan Key Laboratory of Molecular Biology and Biotechnology, College of Life Sciences, Sichuan University, Chengdu, China; 3 Honghe University, Mengzi, China; IRIG-CEA Grenoble, FRANCE

## Abstract

The MADS-box gene family encodes transcription factors with many biological functions that extensively regulate plant growth, development and reproduction. *Erigeron breviscapus* is a medicinal herb used widely in traditional Chinese medicine, and is believed to improve blood circulation and ameliorate platelet coagulation. In order to gain a detailed understanding of how transcription factor expression may regulate the growth of this potentially important medicinal plant, a genome-wide analysis of the MADS-box gene family of *E*. *breviscapus* is needed. In the present study, 44 MADS-box genes were identified in *E*. *breviscapus* and categorized into five subgroups (MIKC, Mα, Mβ, Mγ and Mδ) according to their phylogenetic relationships with the *Arabidopsis* MADS-box genes. Additionally, the functional domain, subcellular location and motif compositions of the *E*. *breviscapus* MADS-box gene products were characterized. The expression levels for each of the *E*. *breviscapus* MADS-box (*EbMADS*) genes were analyzed in flower, leaf, stem and root organs, and showed that the majority of *EbMADS* genes were expressed in flowers. Meanwhile, some MADS genes were found to express high levels in leaf, stem and root, indicating that the MADS-box genes are involved in various aspects of the physiological and developmental processes of the *E*. *breviscapus*. The results from gene expression analysis under different pollination treatments revealed that the MADS-box genes were highly expressed after non-pollinated treatment. To the best of our knowledge, this study describes the first genome-wide analysis of the *E*. *breviscapus* MADS-box gene family, and the results provide valuable information for understanding of the classification, cloning and putative functions of the MADS-box family.

## Introduction

MADS-box gene family, one of the most extensively studied transcription factor families, are involved in developmental control and signal transduction in eukaryotes [1]. These genes have been identified in fungi [2], animals [3] and plants [4]. Members of the MADS-box gene family possess a conserved N-terminal DNA-binding domain, of approximately 60 amino acids, which binds to CArG boxes [5]. This MADS domain is named after several of its earliest members: yeast Mini chromosome maintenance 1 (MCM1) [2]; *Arabidopsis thaliana* AGAMOUS (AG) [5]; snapdragon DEFICIENS (DEF) [4] and human Serum response factor (SRF) [3]. The MADS-box transcription factors were initially identified as floral organ identity-determination genes [4], and play important roles in plant development [6, 7, 8, 9] especially reproduction [10]. Later reports have shown that members of this gene family also regulate other processes such as fruit development [11], embryogenesis [12] and vegetative organ development [13], suggesting a diverse role for this gene superfamily [14]. Parenicova et al. [15] have reported the functions of MADS-box genes in *A*. *thaliana*. For example, the MADS-box genes of SOC1 (Suppressor of Overexpression of Constants1), FLC1 (Flowering Locus C1), AGL24 (AGAMOUS-Like24), MAF1/FLM (MADS Affecting Flowering1/ Flowering Locus M) and SVP (Short Vegetative Phase) affect flowering time; the genes of AP1 (Apetala1), FUL (Fruitfull) and CAL (Cauliflower) determine the identity of floral meristem; these genes are related to floral organogenesis, such as AP1, SEP1 to SEP3 (Sepallata), AP3 (Apetala3), PI (Pistillata) and AG (Agamous). SHP1, SHP2 (Shatterproof) and FUL regulate fruit formation while TT16 (Transparent Testa16) influence seed pigmentation and endothelium development [15]. Based on characteristics such as gene structure, encoded protein secondary structure and phylogenetic relationship, the plant MADS-box gene family can be divided into two major lineages: type I and type II [16]. This diversity is generated by an ancestral gene duplication event [17]. The plant type II genes, which possess the highly conserved MADS domain, have been extensively studied over the last decade. These genes are also termed the MIKC type genes due to the four characteristic functional domains: the most conserved MADS (M) DNA binding domain [18], the less well conserved intervening (I) domain which is crucial for the formation of DNA dimers [19], the keratin (K) domain mediating protein-protein interactions [20] and the C-terminus (C) domain for regulating transcription activation [21]. Among the four domains, the K domain is very important to the evolution and functional diversify of the type II MADS-box genes in plants [22]. Conversely, the type I MADS-box gene subfamily in plants has remained largely unexplored [23]. Compared with type II genes, type I MADS-box genes have a relatively simple gene structure and lack the K domain. Furthermore, the type I MADS-box genes contain a highly conserved SRF-like MADS domain. In *A*. *thaliana*, type I genes are mainly divided into four subgroups: Mα, Mβ, Mγ and Mδ, based on the phylogenetic relations of the conserved MADS-box domain [15]. The Mδ group is closely related to MIKC* class [24].

With the development of high-throughput sequencing technology, the available whole genome sequences for individual species has expanded exponentially, allowing the systematic study of the expression of key genes and gene families comprehensively during plant growth and development. So far, genome-wide analysis of MADS-box genes have been reported in *A*. *thaliana* [25], *Populus trichocarpa* [26], *Oryza sativa* [10], *Prunus mume* [27], *Brassica rapa* [1], *Malus domestica* [28], *Gossypium hirsutum* [29], *Beta vulgaris* [30], *Sesamum indicum* [31], *Vitis vinifera* [32], *Cucumis sativu*s [33], among others. In *A*. *thaliana*, 108 MADS-box genes have been identified, with functions for nearly half of them having been described [6]. In addition, there are 32, 34, 41, 57, 75, 80, 105, 160 and 207 MADS-box genes in *V*. *vinifera*, *B*. *vulgaris*, *C*. *sativus*, *S*. *indicum*, *O*. *sativa*, *P*. *mume*, *P*. *trichocarpa*, *B*. *rapa* and *G*. *hirsutum*, respectively. In plants, MADS-box genes widely participate in the development of the roots, leaves, flowers and fruits. For example, Tian et al. [28] cloned MdMADS5 gene from apple, which displayed high homology with AP1 from *A*. *thaliana* and could make the flowering time of *A*. *thaliana* advance, inflorescence shorten and cluster leaves decrease after the gene was transferred into *A*. *thaliana*. The *PpMADS11*, *12* and *19* genes of peach all showed expression profiles in stamen, petal and other floral organs [34], while in cucumber, *CUM26* gene was found to play an important role in the development of petals and stamens [35].

*Erigeron breviscapus*, also known as dengzhanhua in Chinese, is a perennial herb in the *Erigeron* genus of the Compositae (Asteraceae) family. It has a beautiful flower which is comprised of yellow disk-like florets and multiple surrounding blue to purple ray florets [36]. The plant is endemic to southwestern China and grows in mid-altitude mountains, subalpine open slopes, grasslands and forest margins from 1000 m to 3500 m [37]. As an important Chinese traditional medicinal plant, *E*. *breviscapus* has been widely used to treat various diseases [38, 39, 40]. Recent studies on *E*. *breviscapus* have focused on characterizing the chemical components [41], pharmacological activities [42, 43] and germplasm resources [44, 45]. However, little is known about how growth and development of *E*. *breviscapus* is regulated at the molecular level. The recent generation of the *E*. *breviscapus* whole genome sequence makes a genome-wide analysis of MADS-box genes in *E*. *breviscapus* possible [36].

In this study, we identified 44 MADS-box genes from the *E*. *breviscapus* genome, analyzed their phylogenetic relationships and defined the conserved motifs. To investigate the underlying molecular mechanisms of MADS-box protein function, we performed a protein-protein interaction network analysis of the MADS-box gene products. In addition, according to the work of Yang et al. [36] and Zhang et al. [46], we analyzed the expression patterns of the *E*. *breviscapus* MADS-box genes in four tissues (flower, leaf, stem and root) and three pollination treatments (self-pollinated, cross-pollinated and non-pollinated), and verified by qRT-PCR. To the best of our knowledge, this extended analysis is the first comprehensive study of the MADS-box gene family in *E*. *breviscapus* and provides valuable information for understanding the classification, cloning expression and analysis of putative functions of this family. The study will also broaden our insight into the functional evolution of the MADS-box genes in plants.

## Materials and methods

### Identification of MADS-box genes in *E*. *breviscapus*

The genomic and protein sequences of *E*. *breviscapus* were downloaded from the National Center for Biotechnology Information (NCBI; http://www.ncbi.nlm.nih.gov/). The sequences of the *A*. *thaliana* MADS-box family were retrieved from the *Arabidopsis* Information Resource (TAIR; http://www.Arabidopsis.org/) [15].

To identify all candidate MADS-box genes in *E*. *breviscapus*, a local BLASTP search with a threshold e-value of 1e−10 was performed using *A*. *thaliana* MADS-box protein sequences as query sequences [47]. The identity and cover region (more than 50%) were used as filter criteria to eliminate improper MADS-box genes. Subsequently, to further verify the reliability of the selected sequences, the Pfam database (http://pfam.sanger.ac.uk/search) was used for domain analysis to ensure the presence of the MADS-box domain in each candidate EbMADS protein [48].

### Multiple sequence alignment and phylogenetic analysis between *E*. *breviscapus* and *A*. *thaliana*

Multiple sequence alignments of MADS-box proteins in *E*. *breviscapus* and *Arabidopsis* were performed using ClustalW program in MEGA X 10.1 software with the default settings [49]. The aligned sequences were saved as a .meg extension by choosing Export Alignment from the Data menu and export the file. Choose Open a File/Session from the file menu and open the .meg aligned sequences file in MEGA X 10.1’s main window. A phylogenetic tree was constructed by the Test Maximum Likelihood (ML) method using the pairwise deletion option and poisson correction model. In addition, bootstrap values were calculated with 1000 replications to examine the statistical reliability of the result [49, 50]. The resulting phylogenetic tree data was exported as a Newick file to import a tree into the FigTree version 1.4.3 (http://tree.bio.ed.ac.uk/software/figtree/). Polar tree layout was chosen to visualize a ring phylogenetic tree. Taxa and Clade program made the phylogenetic tree aesthetic.

To further confirm the accuracy of the phylogenetic tree, Bayesian analysis was conducted by using Markov chain Monte Carlo (MCMC)method with MrBayes version 3.12 software [51]. The parameters were set as follows: four Markov chains per analysis, run 2 million times with random tree as the starting tree and sample every 100 generations and repeat once. After discarding burn in sample, consensus tree is constructed based on the remaining samples [52, 53].

### Analysis of conserved motifs and physicochemical properties

To identify shared motifs and structural divergences among the proteins encoded by the MADS-box genes, translated MADS-box protein sequences were subjected to MEME (version 4.12.0, http://meme-suite.org/tools/meme) analysis using the default parameters with the exception that the number of motifs was set to seven [54].

The ProtParam online tool (http://web.expasy.org/protparam/) was used to estimate the basic physicochemical properties of the protein, such as the isoelectric point and molecular weight of the gene product for each member of *E*. *breviscapus* MADS-box gene family. Finally, the subcellular localization of 44 MADS-box genes was predicted by four online analysis tools, such as WoLF PSORT Prediction (https://wolfpsort.hgc.jp/?tdsourcetag=s_pcqq_aiomsg), PSORT Prediction tool (https://www.genscript.com/psort.html), Plant-mPLoc server (http://www.csbio.sjtu.edu.cn/bioinf/plant-multi/) and LocTree3 Prediction system (https://rostlab.org/services/loctree3/). The subcellular localization of the MADS-box genes was retained only results which were confirmed by more than one approach.

### Analysis of the protein–protein interaction network

Protein-protein interaction (PPI) data was obtained from the online database STRING (https://string-db.org/cgi/info.pl), an open source software interface for predicting and visualizing complex networks. The data for PPI stored in the database were derived from experimental validation reports in peer-reviewed journals, including the physical interactions and enzymatic reactions found in signal transduction pathways. The PPI data were preprocessed, including removing redundancy and self-loops. Targets with a high confidence score >0.7 were selected to construct the PPI networks [7]. PPI networks are visualized in Cytoscape with the nodes representing the proteins/genes and the edges representing interactions between any two proteins/genes.

### Expression analysis of *Erigeron breviscapus* MADS-box genes

The genome-wide transcriptome data of *E*. *breviscapus* in different tissues and three pollination treatments were obtained from the NCBI SRA databases under Bioproject Accession codes PRJNA352312 [36] and SRA24595 [46]. The raw reads that contained adapters or more than 5% unknown ‘N’ and low-quality bases as identified by CycleQ 30, were removed. After filtering, gene expression levels were normalized using edgeR with FPKM (Fragments Per Kilobase of transcript per Million mapped reads) value [55]. An FPKM filtering cutoff of 1.0 in at least one of the collected samples, was used to determine expressed transcripts. According to the GeneID of 44 *EbMADS* genes in *E*. *breviscapus* expressed transcripts, the expression data of these genes in four different tissues (root, stem, leaf and flower) and three pollination treatments (non-pollination, self-pollination and cross-pollination) were obtained. The expression profiles were displayed in a heatmap generated with the Heatmap Illustrator software (v 1.0.3.7) by the default data normalization parameter (Linear) and clustering method (Average Linkage) [56].

Quantitative real-time PCR (qRT-PCR) was performed to further confirm the reliability of the expression profile results via six selected genes (including *EbMADS1*, *4*, *10*, *13*, *15* and *39*). The genome-wide transcription data of *E*. *breviscapus* were obtained from four young tissues (root, stem, leaf and flower of wild-type *E*. *breviscapus*) and three pollination treatments (harvested at 24 h after non-(Sample T1), self- (Sample T2) and cross-pollination (Sample T3). Total RNA of all collected samples was extracted using the TRIzol Reagent (Takara, Beijing, China) following the manufacturer’s instructions. The qRT-PCR analysis was performed in a Roche detection system (Roche, Switzerland) using SYBR Green assays. 18s RNA was served as the reference gene to normalize the target gene expression and to correct the variation between samples. The gene-specific primers for the qRT-PCR analysis of six selected genes and reference gene were listed in the S1 Table. The reaction conditions were 30 s at 94 °C, 45 cycles of 20 s at 94 °C 20 s at 55 °C, and 30 s at 72 °C. The melting curves were analyzed from 60 °C to 95 °C to observe the specificity of the PCR products. The comparative 2−ΔΔCT method was employed to calculate the relative expression between samples [57]. All calculations were performed using PASW Statistics 18.0 [58].

## Results

### Identification of the MADS-box genes in *E*. *breviscapus*

To identify the members of the *E*. *breviscapus* MADS-box gene family, 108 *Arabidopsis* genes were employed as a query to search against the *E*. *breviscapus* database by the BLAST programs. In total, 44 putative MADS-box genes were identified in *E*. *breviscapus* and serially named as *EbMADS1* through *EbMADS44* for convenience (Table 1). Most of the genes contained both SRF-TF domain and K-box domain while some genes coded for either SRF-TF domain or K-box domain. In addition, the results showed that the MADS-box genes varied substantially in the length of the mRNA transcripts and their encoded protein sequences. The length of the 44 *EbMADS* mRNA products ranged from 117 to 981 bp and the length of the translated protein sequences varied from 39 to 327 amino acids (Table 1).

**Table 1:** The basic information of MADS-box family members in *Erigeron breviscapus*.

Nomenclature	Accession number in NCBI	Length of mRNA	Group	Length of protein	Number of domains	Domains	MW (kDa)	PI	Subcellular location
*EbMADS*1	AAO22986	588	Mβ	196	2	SRF-TF K-box	22.98	7.62	Nuclear
*EbMADS*2	CAX65571	459	Mγ	153	1	K-box	17.54	6.34	Nuclear
*EbMADS*3	CAH04879	849	Mγ	283	2	SRF-TF K-box	32.21	8.40	Nuclear
*EbMADS*4	CAA57445	708	Mγ	236	2	SRF-TF K-box	27.24	9.60	Nuclear
*EbMADS*5	AAO22982	489	Mγ	163	2	SRF-TF K-box	18.71	9.93	Nuclear
*EbMADS*6	CAA08802	642	Mβ	214	2	SRF-TF K-box	24.92	9.86	Nuclear
*EbMADS*7	AAV65497	501	Mγ	167	1	K-box	18.61	7.61	Nuclear
*EbMADS*8	EOX92192	981	Mδ	327	1	SRF-TF	37.47	5.72	Nuclear
*EbMADS*9	ADU15473	351	Mβ	117	1	K-box	14.13	9.83	Nuclear
*EbMADS*10	ACV86637	126	Mγ	42	1	SRF-TF	4.84	11.61	Nuclear
*EbMADS*11	BAL14660	711	Mγ	237	2	SRF-TF K-box	27.63	8.46	Nuclear
*EbMADS*12	BAK09621	501	Mγ	167	1	K-box	19.04	8.23	Nuclear
*EbMADS*13	XP_002327838	378	Mα	126	1	SRF-TF	14.12	5.71	Nuclear
*EbMADS*14	CAX65661	744	Mγ	248	2	SRF-TF K-box	28.27	9.90	Nuclear
*EbMADS*15	EOX92192	981	Mδ	327	1	SRF-TF	37.44	5.72	Nuclear
*EbMADS*16	CBI15681	285	Mγ	95	1	K-box	11.28	10.54	Nuclear
*EbMADS*17	AAO22980	771	Mγ	257	2	SRF-TF K-box	29.76	10.05	Nuclear
*EbMADS*18	XP_002278239	417	Mγ	139	1	K-box	16.03	4.49	Nuclear
*EbMADS*19	AAV65497	501	Mγ	167	1	K-box	18.61	7.61	Nuclear
*EbMADS*20	ADU56833	672	Mβ	224	2	SRF-TF K-box	25.14	5.52	Nuclear
*EbMADS*21	ADU56833	672	Mβ	224	2	SRF-TF K-box	25.14	5.52	Nuclear
*EbMADS*22	Q9ATE5	765	MIKC	255	2	SRF-TF K-box	29.68	7.16	Nuclear
*EbMADS*23	ACH61565	315	Mγ	105	1	SRF-TF	12.32	11.10	Nuclear
*EbMADS*24	AGQ04483	147	Mγ	49	1	SRF-TF	5.88	10.28	Nuclear
*EbMADS*25	AFA37965	705	Mβ	235	2	SRF-TF K-box	26.28	5.08	Nuclear
*EbMADS*26	EPS59489	117	Mγ	39	1	SRF-TF	4.57	11.29	Nuclear
*EbMADS*27	ACV74250	675	Mβ	225	2	SRF-TF K-box	25.41	5.23	Nuclear
*EbMADS*28	CCO61905	645	MIKC	215	2	SRF-TF K-box	24.37	6.78	Nuclear
*EbMADS*29	CBX45125	438	Mγ	146	1	K-box	17.22	8.21	Nuclear
*EbMADS*30	ADK94172	399	Mγ	133	1	K-box	15.22	6.27	Nuclear
*EbMADS*31	BAN19212	654	Mγ	218	2	SRF-TF K-box	25.24	8.20	Nuclear
*EbMADS*32	CAX65663	447	Mγ	149	2	SRF-TF K-box	17.28	10.27	Nuclear
*EbMADS*33	CBI28594	348	Mβ	116	1	SRF-TF	13.2	10.11	Nuclear
*EbMADS*34	XP_004170688	129	Mγ	43	1	SRF-TF	5.00	10.96	Nuclear
*EbMADS*35	AFO10123	516	MIKC	172	2	SRF-TF K-box	20.02	10.02	Nuclear
*EbMADS*36	CAA57445	708	Mγ	236	2	SRF-TF K-box	27.24	9.60	Nuclear
*EbMADS*37	AAO22986	588	Mβ	196	2	SRF-TF K-box	22.81	9.60	Nuclear
*EbMADS*38	AAO18231	441	Mβ	147	2	SRF-TF K-box	17.29	9.97	Nuclear
*EbMADS*39	CCO61905	696	MIKC	232	2	SRF-TF K-box	26.25	6.30	Nuclear
*EbMADS*40	XP_002522085	618	Mα	206	1	SRF-TF	23.21	10.37	Nuclear
*EbMADS*41	BAN19212	654	Mγ	218	2	SRF-TF K-box	25.24	10.37	Nuclear
*EbMADS*42	AAO22986	588	Mβ	196	1	K-box	22.98	7.62	Nuclear
*EbMADS*43	XP_002278584	783	Mβ	261	2	SRF-TF K-box	29.62	10.05	Nuclear
*EbMADS*44	BAN19217	204	Mγ	68	1	K-box	7.86	4.41	Nuclear

The physicochemical properties of the 44 complete MADS-box amino acid sequences from *E*. *breviscapus* were analyzed using ProtParam (Table 1). The results showed that the molecular weight of these EbMADS proteins ranged from 4.57 to 37.47 kDa. Most of the EbMADS proteins exhibited alkaline isoelectric points greater than 7.5, with the highest being 11.61 for *EbMADS10*, while 12 proteins had acidic isoelectric points of less than 6.5, of which *EbMADS44* was the lowest at 4.41. Two proteins, *EbMADS22* and *EbMADS28* had relatively neutral isoelectric points that fell between 6.5 and 7.5.

Further analysis using four protein subcellular location prediction tools was performed to exactly predict the subcellular localization for the products of the *EbMADS* gene family (S2 Table). As the result shown, all MADS-box proteins were most likely to be located in the nucleus, indicating that although the physicochemical properties of MADS-box transcription factors differed greatly, subcellular location was very conservative (Table 1). Altogether, the results suggest that EbMADS proteins, as transcription factors, play a transcriptional regulatory role directly in the nucleus, consistent with the characteristics of the MADS-box family as transcription factors that regulate transcription of nuclear genomic DNA.

### Classification and phylogenetic analysis of the *EbMADS and AtMADS* gene families

**Fig 1:**
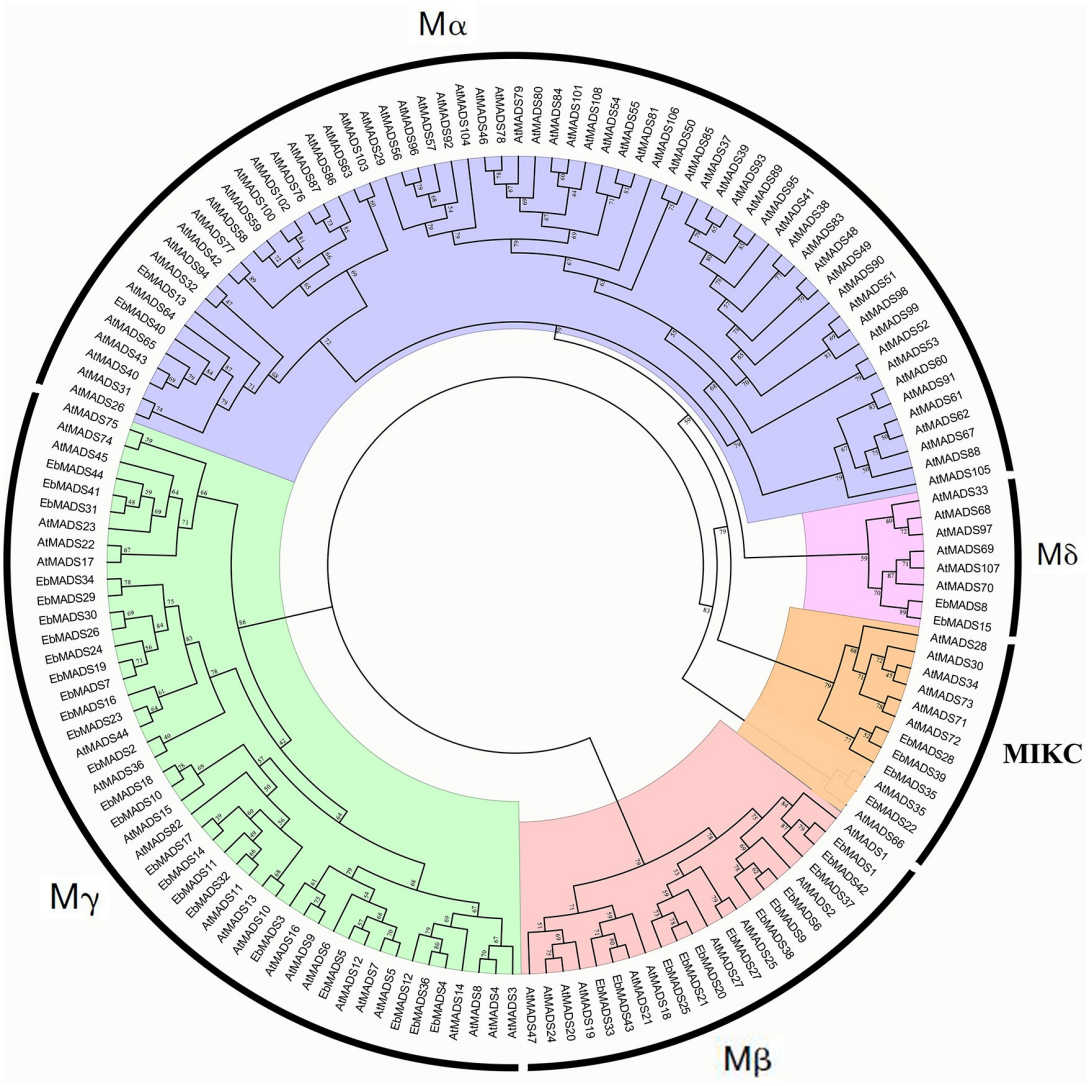
The phylogenetic tree construction of MADS-box genes in *E*. *breviscapus* and *A*. *thaliana*. Maximum Likelihood (ML) phylogenetic tree of all detected MADS-box genes was constructed, using MEGA X 10.1 program with bootstrap analysis (1000 replicates). MADS-box genes in the phylogenetic tree were clustered into five distinct groups (Groups Mα, M β, M γ, M δ and MIKC).

To investigate the evolutionary relationship between *E*. *breviscapus* MADS-box genes in detail, we performed multiple sequence alignments and generated a phylogenetic tree for MADS-box proteins from *E*. *breviscapus* and *A*. *thaliana*. The phylogenetic tree was constructed on the basis of the consistency of Maximum Likelihood and Bayesian phylogenetic tree. In our study, the 44 *EbMADS* genes were classified into functional groups according to *A*. *thaliana* MADS-box genes that had been extensively studied (Fig 1) [15]. *EbMADS* members could be divided into two groups: type I and type II. According to the *A*. *thaliana* phylogenetic relationships, type I proteins could be further divided into three subfamilies as follows: Mα, Mβ and Mγ whereas the type II group contained Mδ and MIKC proteins. As shown in Fig 1, among the 38 type I members in *E*. *breviscapus*, two were grouped as Mα, 12 as Mβ, 24 as Mγ. However, only two Mδ and four MIKC proteins comprised the *EbMADS* type II proteins, such as *EbMADS8*, *15*, *22*, *28*, *35* and *39*. The phylogenetic tree and the website information of *Arabidopsis* MADS-box Transcription Factor Gene Family (https://www.arabidopsis.org/browse/genefamily/MADSlike.jsp) further explained the relationships of the *EbMADS* genes classification by function and structure. For example, *EbMADS13* and *EbMADS40* of the Mα group, *EbMADS8* and *EbMADS15* of the Mδ group, *EbMADS20*, *21*, *25*, *33* and *43* of Mβ group and *EbMADS2*, *3*, *7*, *10*, *16*, *18*, *19*, *23*, *24*, *26*, *29*, *30* and *34* of the Mγ group were all AGL subgroup indicating these affected flowering time. The group of Mγ and SOC1 including *EbMADS31*, *41* and *44*, Mβ and SVP group embodying *EbMADS27* and the group of MIKC and MAF containing *EbMADS28*, *35* and *39* were relevant to the flowering time. Six MADS-box genes of Mβ group which EbMADS1, *37* and *42* pertaining to PI subgroup and *EbMADS6*, *9* and *38* belong to AP3 subgroup and *EbMADS5* of the Mγ and SEP group were related to floral organogenesis. The Mγ and FUL group including *EbMADS11*, *14*, *17* and *32* affected the determination of floral meristem identity while the *EbMADS22* of MIKC and TT16 group played an important role in seed pigmentation and endothelium development. *EbMADS4*, *12* and *36* in Mγ group were belong to unknown subgroups.

### Conserved motifs analysis of *EbMADS* gene families

**Fig 2:**
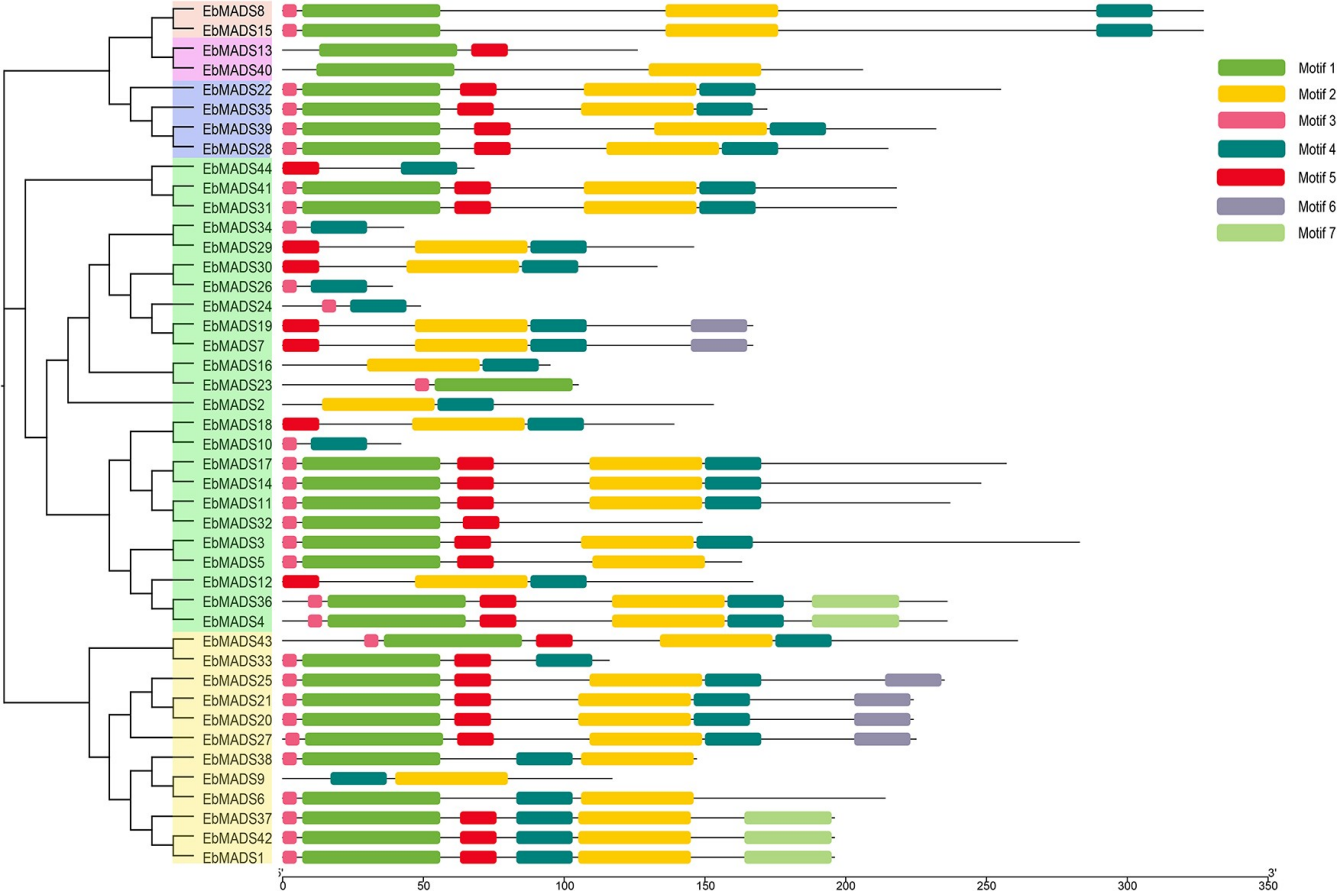
Motif analysis of MADS-box gene family in *Erigeron breviscapus*. Motif compositions: protein sequences are indicated by thick gray lines, and the conserved motifs are represented by different colored boxes. The length (amino acids) of the protein and motif can be estimated using the scale bar at the bottom.

To assess the diversity and similarity of motif composition among the different *E*. *breviscapus* MADS-box genes, the MEME tool was employed to identify motifs within the 44 MADS-box protein sequences. A total of seven conserved motifs (denoted motifs 1–7; Fig 2) were identified in the MADS-box proteins and their consensus sequence information and logo are displayed in Table 2 and S1 Fig respectively. Given the phylogenetic tree and conserved motifs, we note that the *EbMADS* genes clustered in the same subgroup shared substantially consistent conserved motifs, which indicates that members of the same subgroup might possess functional similarities. Mδ-clade (*EbMADS8* and *EbMADS15*) and MIKC-clade (*EbMADS22*, *EbMADS35*, *EbMADS39* and *EbMADS28*) proteins of the type II family contained MADS domains with similar motif compositions. Both clades contained motif1, motif 2, motif 3 and motif 4, while the MIKC subgroup also included motif 5. Conversely, members of the type I MADS family displayed quite different motif composition. Mα, consisting of *EbMADS13* and *EbMADS40*, only had two motifs, either motifs 1 and 5 or motifs 1 and 2. The MADS domains of the majority of the Mβ subfamily contained motif 1, motif 3 and motif 4 except for *EbMADS9*, which had neither motif 1 nor motif 6. The Mγ clade had the most members [29] and showed a complex motif profile. For example, eight gene members all coded for two varied motifs. The remaining gene domains contained at least three motifs, with most having motif 4 in the MADS domain. According to the homology comparison annotation in *A*. *thaliana*, motif 1 was related to DNA binding and motif 3 was found to concern with nuclear localization, which further illustrated the nuclear location of the *EbMADS* gene family. The functions of other motifs were unknown. Furthermore, the seven motifs and corresponding logos of *A*. *thaliana* were analyzed (S2 Fig). Each capital letter represented an amino acid in the motif logos. Same amino acid (or same capital letter) at the same position, suggesting the frequencies of amino acids used by motifs were conservative. As shown in the S1 and S2 Figs, motif distribution in *A*. *thaliana* was more conservative than *E*. *breviscapus*. However, the frequencies of amino acids used by motifs were not very conservative in *A*. *thaliana*, which was similar with *E*. *breviscapus*.

**Table 2:** The information of motif found in MEME.

Motifs	Conserved amino acid sequences	E-value	Sites	Width
1	IKRIENNTNRQVTFSKRRNGLLKKAHELSVLCDAEVALIVFSSTGKLYEY	1.0e-944	30	50
2	RHLLGEDLGGLSLKELEQLEQQLEDGLSRVRSRKDQLMLEQ	7.3e-317	35	41
3	MGRGKI	5.6e-067	33	6
4	IENLQEKEKKLKEENEGLKKK	5.6e-072	40	21
5	MEDILERYQRHSKN	4.4e-057	31	14
6	SGPPPEDDGSDTSLKLGLPFS	5.5e-031	6	21
7	QQSEMNTMGEYQNHQPFSFRVQPLQPNLMERI	1.8e-034	5	32

### Analysis of EbMADS protein function link network

**Fig 3:**
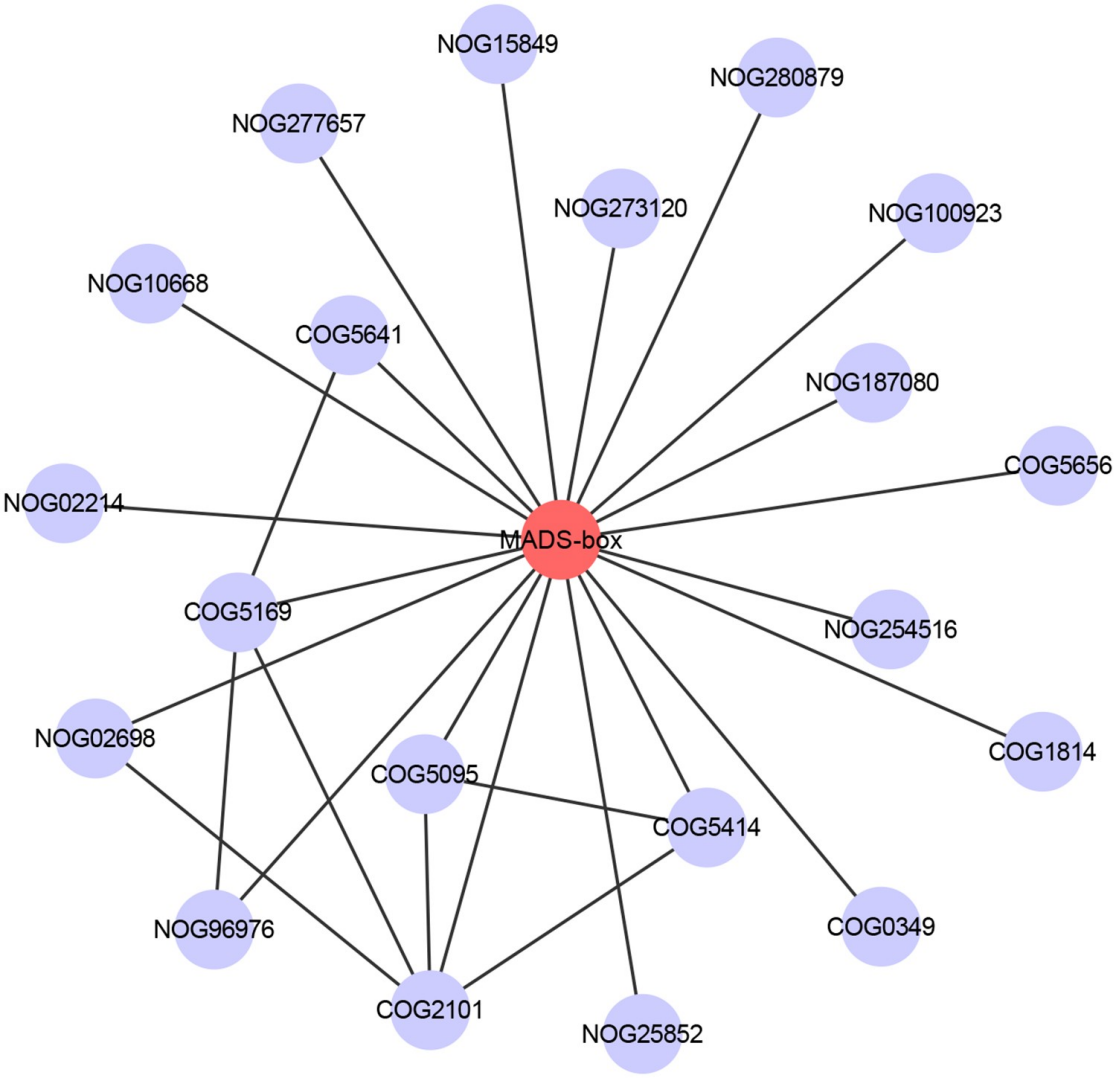
The analysis of EbMADS protein function link network. The PPI data were preprocessed, including removing redundancy and self-loops. Targets with a high confidence score >0.7 were selected to construct the PPI networks. PPI networks are visualized in Cytoscape with the nodes representing the proteins/genes and the edges representing interactions between any two proteins/genes.

To investigate the potential molecular mechanisms of *E*. *breviscapus* MADS-box proteins, the protein patterns stored in the STRING database were used to construct the PPI network. From the results, we found that the EbMADS proteins exhibited a protein-protein interaction with 20 other proteins (Fig 3). Among the co-expression proteins, COG2101 (TATA-box binding protein, component of TFIID and TFIIIB), COG5169 (Heat shock transcription factor), COG5095 (Transcription initiation factor TFIID, subunit TAF6) and COG5414 (TATA-binding protein-associated factor) featured prominently in the protein-protein network, indicating that those proteins are vital to maintaining the protein interactions in the network. Moreover, NOG02698, NOG96976 and COG5641 (GATA Zn-finger-containing transcription factor) played a pivotal role in the network. While most of the key contacts in the PPI network are involved in transcriptional regulation, the function of NOG02698 and NOG96976 are unclear. Additional interactions were noted with non-transcription related proteins, such as COG5656 (Importin, protein involved in nuclear import) and COG0349 (Ribonuclease D). The results presented in this study have provided a way to identify the key proteins which could interact with EbMADS proteins, detailed information this PPI network are listed in S3 Table.

### Tissue specific expression profiles for *E*. *breviscapus* MADS-box genes

**Fig 4:**
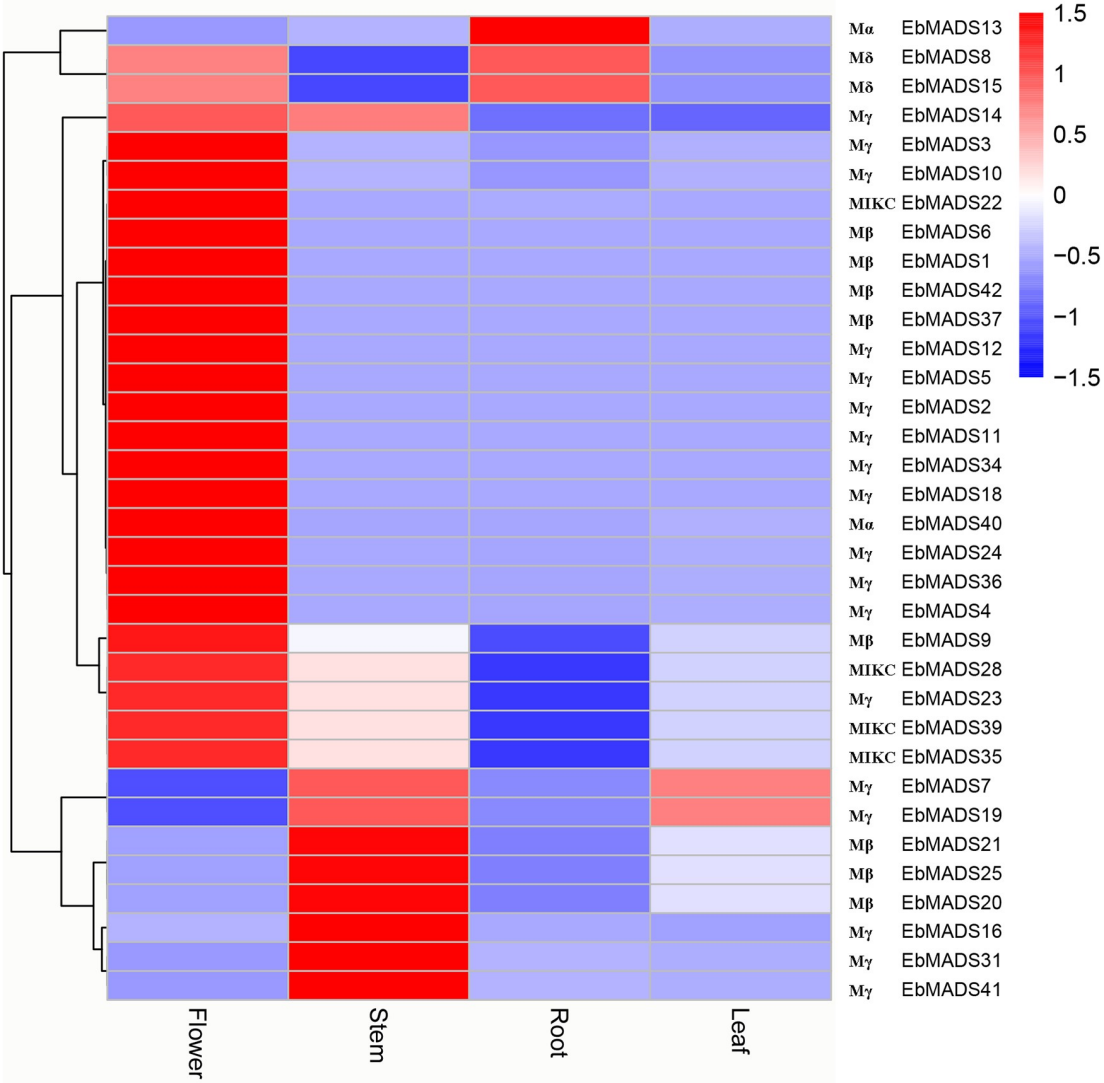
Expression profiles for *E*. *breviscapus* MADS-box genes in different tissues. The tissues included leaf, root, stem, and flower. Gene expression levels were calculated using the FPKM method. The bar at the right of each heat map represents relative expression values, thereby blue color representing low level expression, white shows medium level expression and red signifies high level expression.

MADS-box genes are expressed in different plant organs, such as the vegetative organ roots, stems, leaves, reproductive organs, fruits and seeds, and play important regulatory roles in plant development, growth and reproduction [59, 60]. In order to gain insight into the tissue specific *E*. *breviscapus* MADS-box gene expression pattern and to elucidate their potential roles in tissue development, we utilized transcriptome data derived from Illumina RNA-Seq reads generated by Yang et al. [36]. The transcript abundance from each of the 34 *EbMADS* genes in four different tissues, including root, stem, leaf and flower, were analyzed and compared. As shown in Fig 4, *EbMADS* genes were expressed in all four *E*. *breviscapus* tissues studied. The majority of *EbMADS* genes showed high expression levels in flowers, consistent with MADS-box genes being originally identified as floral organ regulatory genes [4]. For instance, *EbMADS1*, *37* and *42* of PI subgroup and *EbMADS6*, *9* and *38* of AP3 subgroup were B-class genes in the ABC model, related to floral organogenesis. *EbMADS8* and *EbMADS15*, belong to Mδ group, were expressed in both flowers and roots to similar levels, while the expression of *EbMADS7* and *EbMADS19* genes of Mγ group were noted in both leaves and roots. Three MIKC-type MADS-box genes, including *EbMADS28*, *EbMADS35* and *EbMADS39*, were all expressed in both flowers and stems, however the transcript levels were less in stems than in flowers. Interestingly, ten *EbMADS* genes showed no expression in any of the tissue expression data studied. These genes played an important role in other plant tissues such as fruit development could be speculated [8]. To further confirm the expression profiles of the MADS-box genes in four tissues, six *EbMADS* genes were selected for qRT-PCR analysis (S3 Fig). *EbMADS1*, *4*, *10* and *39* showed high expression levels in flower while *EbMADS13* had maximal expression in root. *EbMADS15* presented high levels of expression in flower and root. The results of qRT-PCR had strong consistency with those of transcriptome analysis.

### Effect of pollination treatment on expression patterns of *E*. *breviscapus* MADS-box genes

**Fig 5:**
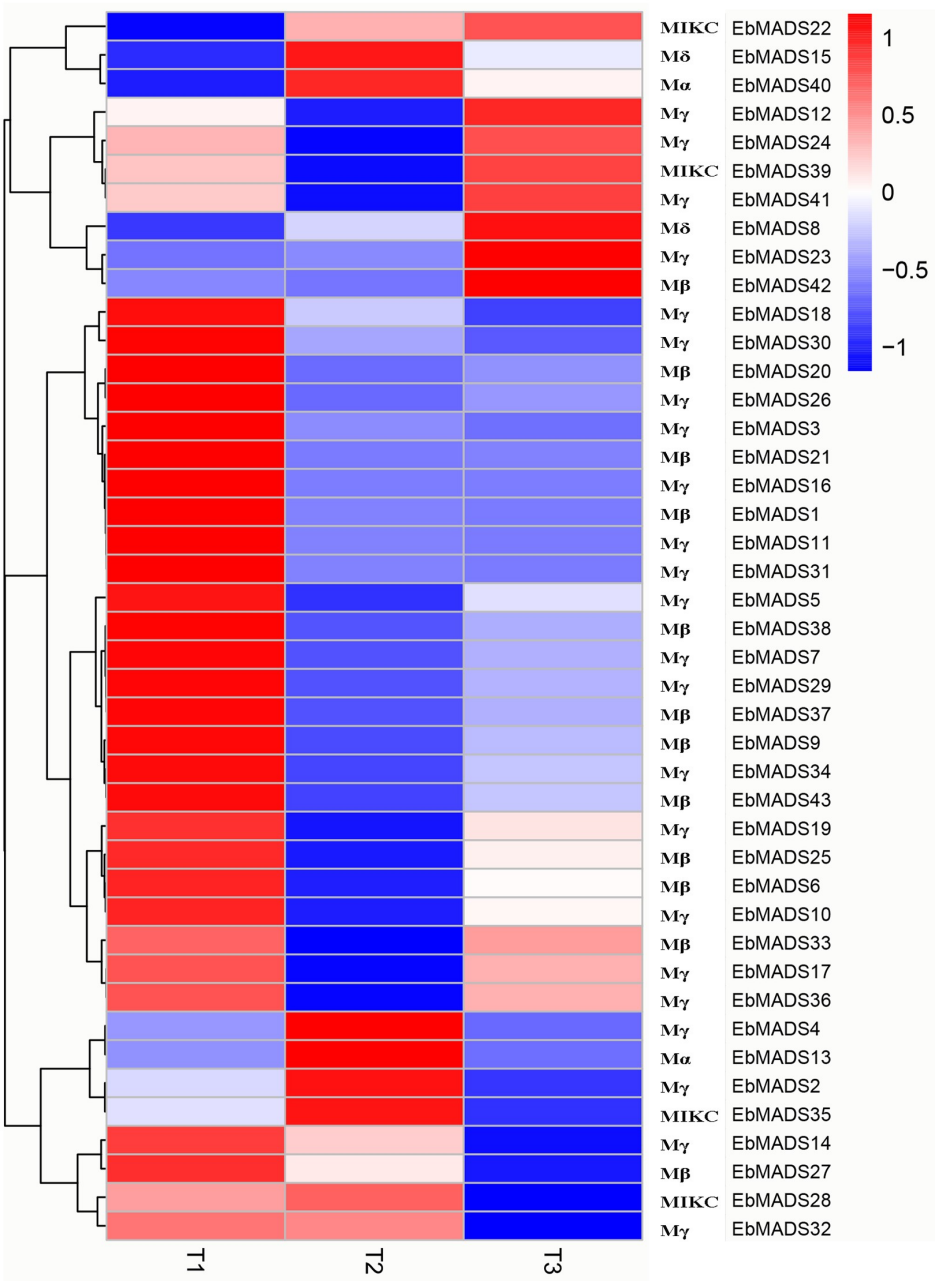
Expression patterns of *E*. *breviscapus* MADS-box genes in three pollination treatments. T1, T2 and T3 indicated samples were respectively forced with non-pollinated, self-pollinated, and cross-pollinated treatment. The bar at the right of each heat map represents relative expression values, thereby blue color representing low level expression, white shows medium level expression and red signifies high level expression.

Self-incompatibility (SI) is an important mating system in many flowering plants, which ensures genetic diversity and is beneficial to plant evolution and adaptation to the environment [61, 62]. As a species of Asteraceae, *E*. *breviscapus* is self-incompatible. To further understand the potential functions of MADS-box genes in *E*. *breviscapus* SI responses, genome-wide transcriptome data, from three different pollination treatments, deposited by Zhang et al. [46], was analyzed. Heatmap representation of the expression profiles of the 43 *EbMADS* genes in non- (Sample T1), self- (Sample T2) and cross-pollination (Sample T3) treatments are shown in Fig 5, revealing that most of the MADS-box genes displayed a broad expression spectrum after non-pollination treatment. From the results, we found that a total of 26 MADS-box members exhibited maximal expression in this data set. Conversely, *EbMADS24*, *EbMADS41*, *EbMADS17*, *EbMADS36* and *EbMADS32* of Mγ subfamily and MIKC subfamily including *EbMADS39* and *EbMADS28* shared the characteristic of having low expression after non-pollination treatment. In the cross-pollination treatment data, eight MADS-box genes displayed high expression levels, with six additional genes, including *EbMADS19*, *EbMADS10*, *EbMADS17* and *EbMADS36* of Mγ group and Mβ group containing *EbMADS25* and *EbMADS33*, showing low expression levels. However, compared with the expression patterns observed for non- and cross-pollination data, expression of the MADS-box genes was significantly down-regulated in self-pollination treatment. For example, while transcript abundances for *EbMADS15*, *EbMADS40*, *EbMADS13*, *EbMADS4*, *EbMADS2* and *EbMADS35* genes were all high, *EbMADS22*, *EbMADS27*, *EbMADS14* and *EbMADS32* showed relatively low transcript abundance. These results showed that MADS-box genes expression may be inhibited during self-pollination, causing the self-incompatibility of *E*. *breviscapus* reproduction. The results of qRT-PCR about the six *EbMADS* genes were significantly corroborated those of transcriptome analysis (S4 Fig). For example, *EbMADS1* and *EbMADS10* showed high expression levels under non-pollination treatment whil*e EbMADS4* and *EbMADS13* had maximal expression levels under self-pollination treatment. The *EbMADS15* mainly expressed under self- and cross-pollination treatments. *EbMADS39* showed higher expression levels in the treatments of non- and cross-pollination.

## Discussion

*E*. *breviscapus* is an important traditional Chinese medicine. At present, it is widely used as raw material ingredients in remedies for cardiovascular and cerebrovascular diseases [63]. Use in the treatment of diabetes, nephropathy and senile diseases are also common [64]. In addition, it has been reported that *E*. *breviscapus* has an anti-cancer effect [65]. MADS-box genes, important transcription factors in plants, play significant roles in plant growth and development process. In flowering plants, MADS-box proteins have important biological significance in a wide range of processes, including the control of flowering time, the determination of floral meristem, the identification of floral organ, fruit development, endosperm development and nutritional development [59]. Recently, as successive advances are made in the genome sequencing of various species, the knowledge of the role of MADS-box genes in plant development is deepening. Increasingly, MADS-box gene families in numerous plants have been identified and analyzed from sequenced species using genome-wide bioinformatics analysis tools. MADS-box gene family identification and evolution analysis of *A*. *thaliana* [15], *O*. *sativa* [10], *P*. *trichocarpa* [26], *V*. *vinifera* [32], *Cucumis sativus* [66], *Glycine max* [9], *P*. *mume* [27], *B*. *rapa* [1], *M*. *domestica* [28] and *G*. *hirsutum* [29] have been successively completed. In the current study, these bioinformatics tools have been used to identify and predict the function of the *MADS* genes in the complete genome sequence of *E*. *breviscapus*.

MADS-box genes contain a highly conserved MADS-box domain composed of about 60 amino acids. A phylogenetic analysis of MADS-box genes from *A*. *thaliana*, fungi and animals performed by Alvarez-Buylla et al. [67] showed that the MADS-box genes underwent a gene duplication before the divergence of plants and animals, bringing about type I (SRF-like) and type II (MEF2-like) lineage. According to the MADS-box gene structure, duplication and motif analysis of *A*. *thaliana*, Parenicova et al. [15] suggested the type-I and type-II MADS-box genes can be further divided into five distinct subgroups, named Mα, Mβ, Mγ, Mδ and MIKC. Phylogenetic analysis of the MADS-box gene family in rice, determined that type I genes contained four subfamilies Mα, Mβ, Mγ and Mδ; while type II consisted of MIKC subgroups [10]. Of the 146 MADS-box genes identified in apple, 82 members could be unambiguously classified as MIKC type II, whereas the remaining 64 members were classified as type I (including Mα, Mβ, Mγ and Mδ) [28]. However, as shown in the phylogenetic dendrogram for *P*. *mume* MADS-box genes, while the genes of Mα, Mβ and Mγ subfamilies were type I MADS-box genes, the Mδ clade showed a similar phylogenetic tree to the type II genes [27]. This is consistent with the phylogenetic analysis of three cotton species *(Gossypium raimondii*, *Gossypium arboreum* and *Gossypium hirsutum*), determined that the type I lineage contained Mα, Mβ and Mγ groups while the type II lineage was comprised of both Mδ and MIKC [29]. Interestingly, studies of some species showed the absence of the Mδ subfamily. In this study, comparison of the phylogenetic trees of *A*. *thaliana* and *E*. *breviscapus* determined that *E*. *breviscapus* MADS-box genes were subdivided into five groups, including Mα, Mβ, Mγ, Mδ and MIKC. The MADS-box domains of Mα, Mβ, Mγ and MIKC were generally conserved, showing similar motif structures. The Mδ gene domains were simple and noted as components of the MIKC gene motifs. The Mδ and MIKC clades were closely related in the phylogenetic tree. Therefore, the type I MADS-box genes were confirmed to consist of three subgroups: Mα, Mβ and Mγ while the Mδ and MIKC clades formed type II MADS-box genes. The classification is similar to *G*. *raimondii*, *G*. *arboreum* and *G*. *hirsutum* [29].

MADS-box genes are widely expressed in plants, and known to be involved in multifarious and important aspects of vegetal development and differentiation. As key players in the regulation of developmental mechanisms at the molecular level, the function of MADS-box genes are extensively observed, not only for the flower organ [68], but also the regulation in fruit [69], root and leaf development [16]. In the present study, we use tissue specific transcriptomic data to compare the expression of *E*. *breviscapus* MADS-box genes in the flowers, stems, roots and leaves. The majority of MADS-box genes of *E*. *breviscapus* shared the same expression patterns in flowers, implying a functional redundancy in this organ and consistent with MADS-box genes originally being identified as flower related genes. The ABC model was widely known to explain the combined functions of A (AP1 and AP2), B (PI and AP3) and C (AG) classes genes to determine the *Arabidopsis* flower organs identity [15]. In *E*. *breviscapus*, there were only B-class genes (*EbMADS1*, *37* and *42* of PI, *EbMADS6*, *9 and 38* of AP3) further suggesting the six MADS-box genes were exactly related to floral organogenesis. In addition, the results of 25 *EbMADS* genes expressed in flowers most agreed with the categories by function, such as *EbMADS28*, *35* and *39* of MAF subgroup related to flowering time, *EbMADS11* and *14* of FUL subgroup and flower meristem identity to be interrelated and *EbMADS5* in SEP subgroup bound up with floral organogenesis. Furthermore, MADS-box genes are expressed in the flowers of many plants. For example, *OsMADS3* controls terminal anther development in rice through regulating ROS homeostasis [70]. Nine out of 18 members of the MADS-box genes in cherry had expression profiles only in flower organs [71]. In *Crocus sativus*, the *CsMADS* genes, belonging to different MADS-box subfamilies direct the formation of floral organs by regulating the development of flower organs in different rounds [72]. In this study, more *EbMADS* genes were expressed in stems than in roots and leaves. Similarly, in sesame plants, *SiMADS* genes were highly expressed in both the flower buds and stem tips, where the first flower appears at the top of stem at the 10 or 12 leaf stage [31]. This may also be the reason why more gene members of *E*. *breviscapus* MADS-box family show specific and efficient expression in plant stems.

Studies have indicated that the MADS-box genes play an important role in the plant pollination process. Yang et al. [36] found the MADS-box gene in *O*. *sativa*, named *OsMADS29*, was highly expressed in development seeds after pollination [73]. The steady state expression of two maize MADS-box genes *ZMM6* and *ZMM27* increased in kernels after pollination [74]. And Ning et al. [75] suggested activated carbohydrate metabolism, cell division and expansion as well as the down-regulation of MADS-box could comprehensively regulate the plant pollination-dependent and parthenocarpic fruit set. *E*. *breviscapus* possesses the pollination system [76]. However, *E*. *breviscapus* is a member of the Asteraceae family, the archetypical plant that displays self-incompatible reproduction [77]. Such self-incompatibility systems widely exist in plants as a mechanism to maintain genetic diversity in their offspring [78]. Self-incompatibility or self-sterility, is the situation where a plant lacks the ability to self-pollinate. To understand the possible role of *EbMADS* genes in the self-incompatibility reaction, we performed an expression profile analysis on transcriptomic data from self-pollination and cross-pollination experiments. The results showed most of the *EbMADS* genes displayed high expression levels in the non-pollinated treatment data, which indicates that MADS-box gene family has a vital impact on *E*. *breviscapus* growth and development. Interestingly, the expression patterns of the genes from cross-pollinated plants were similar to that from non-pollinated plants, suggesting that cross-pollination plays an important role in the natural development progress of the *E*. *breviscapus*. Conversely, only seven genes (including *EbMADS22*, *EbMADS15*, *EbMADS40*, *EbMADS4*, *EbMADS13*, *EbMADS2* and *EbMADS35*) displayed high expression levels in the self-pollinated treatment, indicating that MADS-box genes expression may be inhibited during self-pollination suggesting the molecular mechanism that may underlie the self-incompatibility of *E*. *breviscapus*.

## Supporting information

S1 Fig Sequence logos of the *Erigeron breviscapus* conserved motifs. (TIF)

S2 Fig Motif analysis and sequence logos of MADS-box gene family in *Arabidopsis thaliana*. (TIF)

S3 Fig Correlation analysis of qRT-PCR for *E*. *breviscapus* MADS-box genes in different tissues. (TIF)

S4 Fig Correlation analysis of qRT-PCR for *E*. *breviscapus* MADS-box genes in three pollination treatments. (TIF)

S1 Table qRT-PCR Primers used in this study. (DOC)

S2 Table The statistical outputs of MADS-box protein subcellular location by four prediction tools. (DOC)

S3 Table The detailed information of the proteins in the PPI network. (XLS)
