# Supplementary figures and images for: Genome-wide identification and expression profile of the MADS-box gene family in Erigeron breviscapus
Source: PLoS One. 2019 Dec 20;14(12):e0226599. doi: 10.1371/journal.pone.0226599 (PMC6924644; doi:10.1371/journal.pone.0226599)

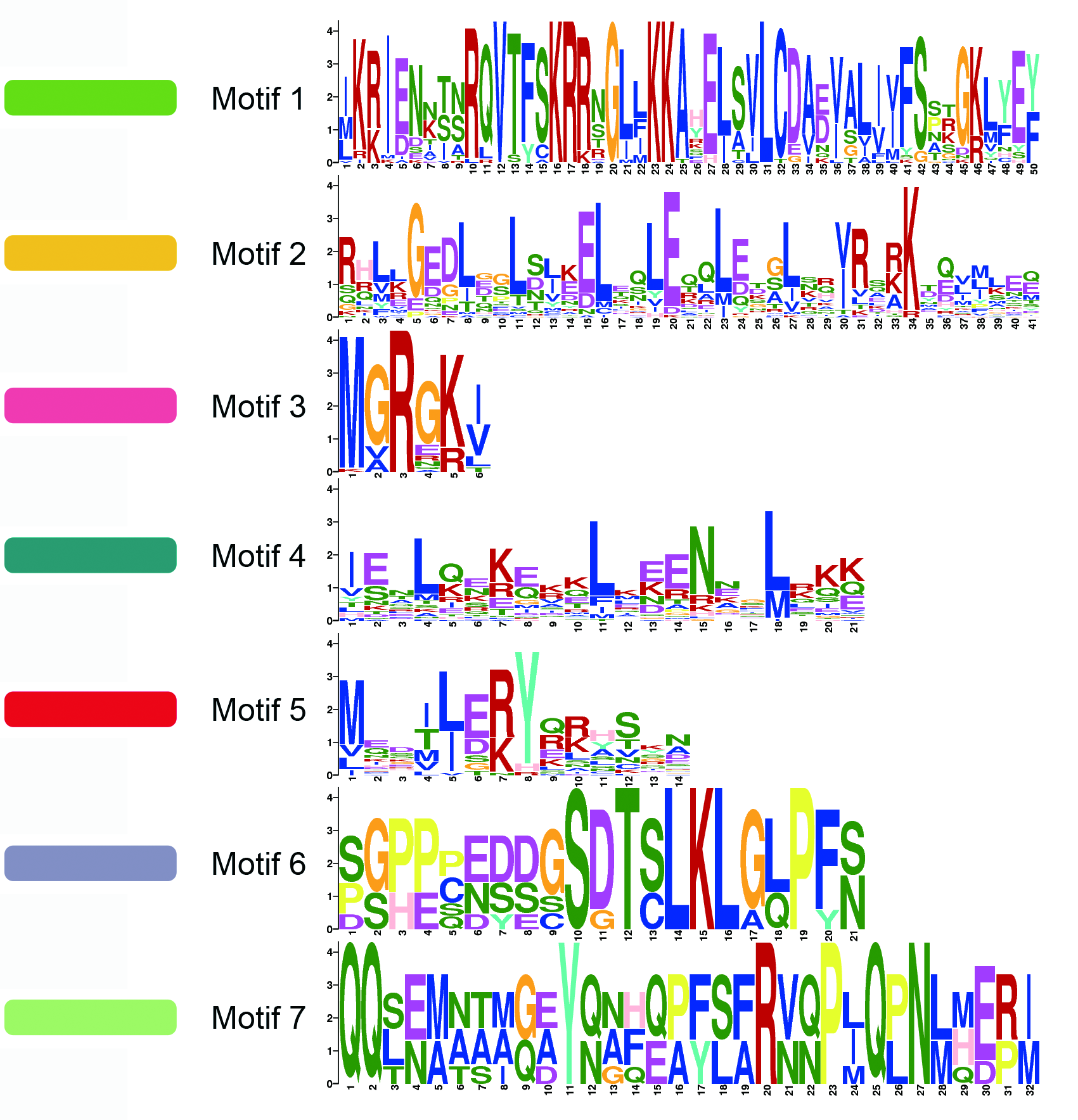

Supplement: S1 Fig — (TIF) [file pone.0226599.s001.tif]

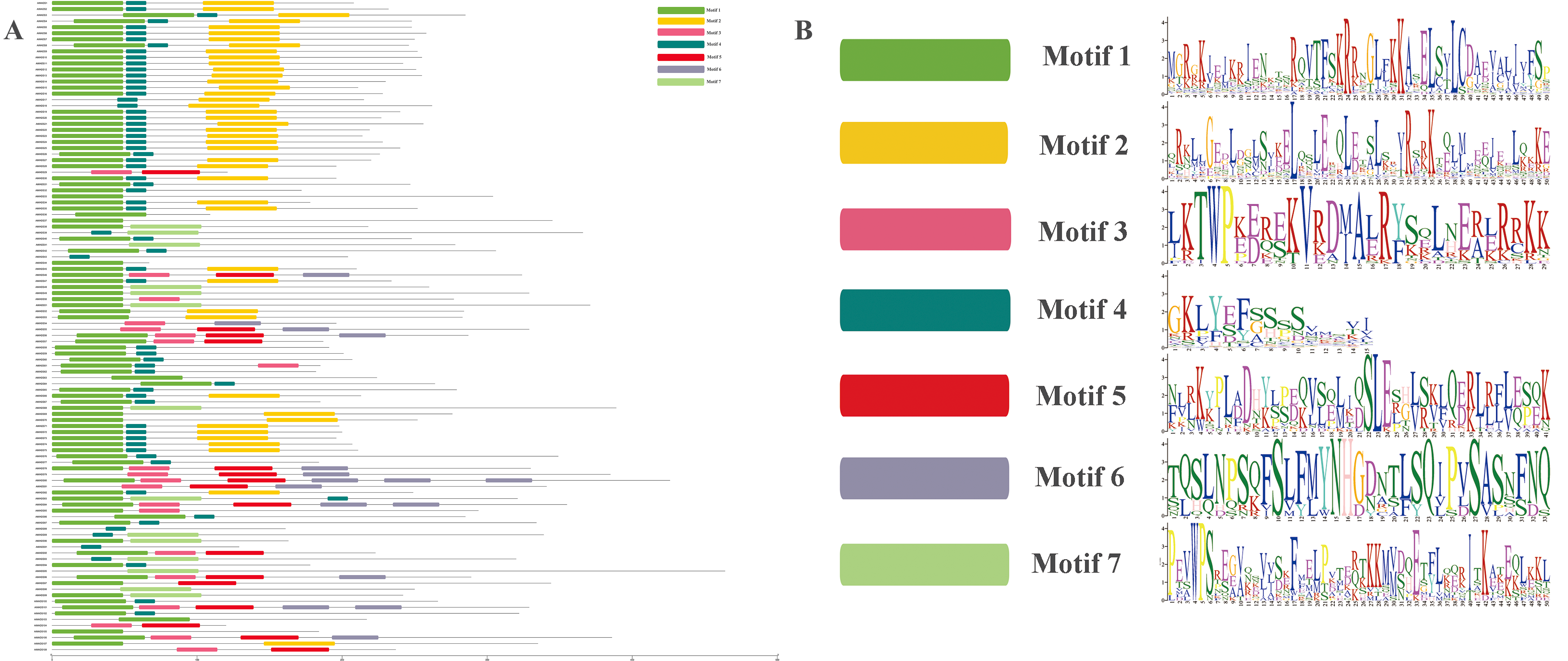

Supplement: S2 Fig — (TIF) [file pone.0226599.s002.tif]

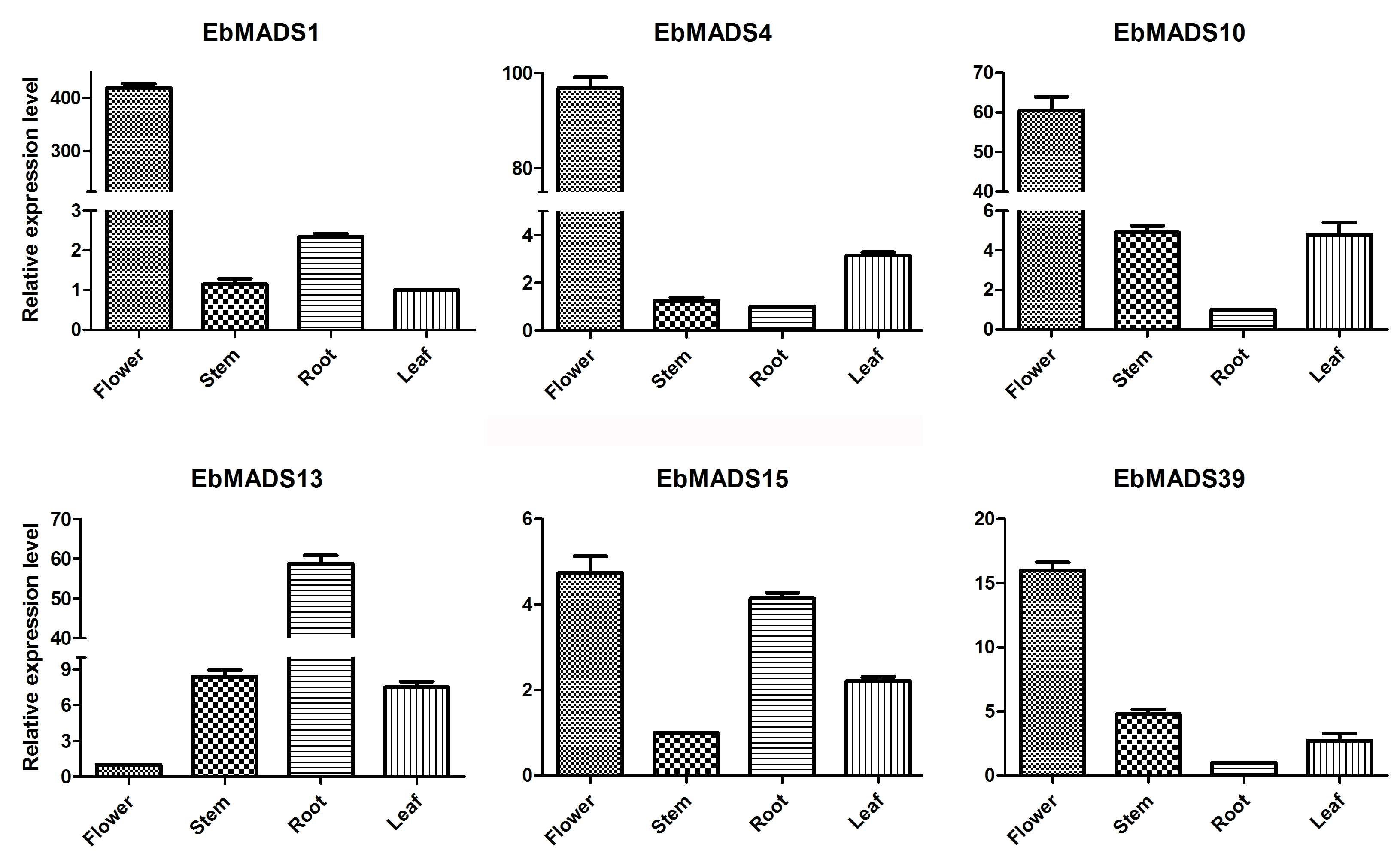

Supplement: S3 Fig — (TIF) [file pone.0226599.s003.tif]

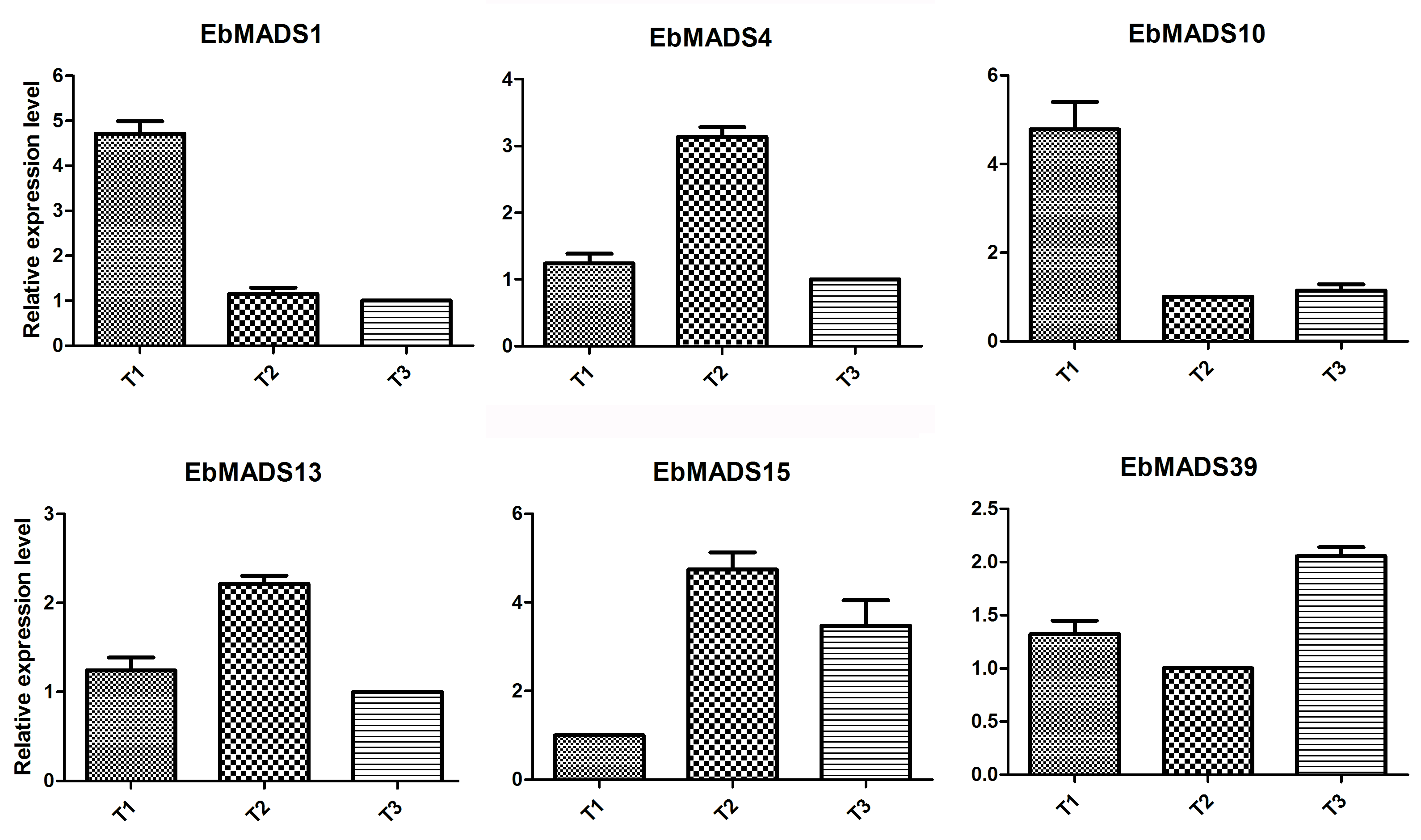

Supplement: S4 Fig — (TIF) [file pone.0226599.s004.tif]
