# Supplementary material for: Genome-wide identification and expression profile of the MADS-box gene family in Erigeron breviscapus
Source: PLoS One. 2019 Dec 20;14(12):e0226599. doi: 10.1371/journal.pone.0226599 (PMC6924644; doi:10.1371/journal.pone.0226599)
Supplement: S1 Table — (DOC) [file pone.0226599.s005.doc]

| **Primer** | **Sequence (5′-3′)** | **Purpose** | **Amplicon size** |
| --- | --- | --- | --- |
| MADS1-F | ATCAAAGGCTGTCTGGGAATAA | qRT-PCR of MADS1 | 110 |
| MADS1-R | ATGCCTAAGCTCAATTTGCATAC | qRT-PCR of MADS1 |
| MADS4-F | AACACAGCACAGGCGTATAA | qRT-PCR of MADS4 | 108 |
| MADS4-R | CTCAGGAATGTAACCAGGAGTG | qRT-PCR of MADS4 |
| MADS10-F | GGGTAGAGGAAAGGTACAGTTAAG | qRT-PCR of MADS10 | 90 |
| MADS10-R | TTCTTCAATAAGCCACCTCTCC | qRT-PCR of MADS10 |
| MADS13-F | CCCATGTGGGCGTATGTATT | qRT-PCR of MADS13 | 93 |
| MADS13-R | GCGATCACGTCTTGGTTACT | qRT-PCR of MADS13 |
| MADS15-F | TCGGAAAGAGGAAGTGTGATTC | qRT-PCR of MADS15 | 95 |
| MADS15-R | CAGGGAATGTGTATGGCTAACT | qRT-PCR of MADS15 |
| MADS39-F | ACAGTGGACAGTTGTTCTACC | qRT-PCR of MADS39 | 97 |
| MADS39-R | CAGACAGCTCCTCGGTATTATTT | qRT-PCR of MADS39 |
| 18S-F | TCTGCCCGTTGCTCTGATG | qRT-PCR of 18S | 130 |
| 18S-R | TCACCCGTCACCACCATAG | qRT-PCR of 18S |
