# Supplementary material for: Genome-wide identification and expression profile of the MADS-box gene family in Erigeron breviscapus
Source: PLoS One. 2019 Dec 20;14(12):e0226599. doi: 10.1371/journal.pone.0226599 (PMC6924644; doi:10.1371/journal.pone.0226599)
Supplement: S2 Table — (DOC) [file pone.0226599.s006.doc]

**Table S2 The statistical outputs of MADS-box protein subcellular location by four prediction tools**

|  | **PSORT Prediction tool** | **WoLF PSORT Prediction** | **Plant-mPLoc server** | **LocTree3 Prediction system** |
| --- | --- | --- | --- | --- |
| EbMADS1 | 91.3 %: nuclear  8.7 %: mitochondrial  >> prediction for 15688001872133 is nuc (k=23) | nucl: 14 | Nucleus | Nucleus: 93% |
| EbMADS2 | 60.9 %: nuclear  34.8 %: mitochondrial  4.3 %: cytoplasmic  >> prediction for 15688000802134 is nuc (k=23) | cyto: 5, mito: 5,  nucl: 2, chlo: 1,  cysk: 1 | Nucleus | Nucleus: 93% |
| EbMADS3 | 95.7 %: nuclear  4.3 %: mitochondrial  >> prediction for 15688044992134 is nuc (k=23) | nucl: 14 | Nucleus | Nucleus: 92% |
| EbMADS4 | 73.9 %: nuclear  17.4 %: mitochondrial  8.7 %: cytoplasmic  >> prediction for15688045602133 is nuc (k=23) | cyto: 7, nucl: 5,  mito: 1, plas: 1 | Nucleus | Nucleus: 89% |
| EbMADS5 | 95.7 %: nuclear  4.3 %: mitochondrial  >> prediction for 15688046082135 is nuc (k=23) | nucl: 13, pero: 1 | Nucleus | Nucleus: 97% |
| EbMADS6 | 87.0 %: nuclear  13.0 %: mitochondrial  >> prediction for 15688046502134 is nuc (k=23) | pero: 11, nucl: 1,  cyto: 1, golg: 1 | Nucleus | Nucleus: 89% |
| EbMADS7 | 69.6 %: nuclear  13.0 %: mitochondrial  8.7 %: peroxisomal  8.7 %: cytoplasmic  >> prediction for 15688046982136 is nuc (k=23) | nucl: 9, chlo: 2,  mito: 2, plas: 1 | Nucleus | Nucleus: 84% |
| EbMADS8 | 69.6 %: nuclear  30.4 %: mitochondrial  >> prediction for 15688048492134 is nuc (k=23) | nucl: 14 | Nucleus | Nucleus: 87% |
| EbMADS9 | 87.0 %: nuclear  4.3 %: peroxisomal  4.3 %: vesicles of secretory system  4.3 %: mitochondrial  >> prediction for 15688048802134 is nuc (k=23) | nucl: 7, cyto: 3,  chlo: 1, plas: 1,  extr: 1, cysk: 1 | Nucleus | Nucleus: 87% |
| EbMADS10 | 82.6 %: nuclear  17.4 %: mitochondrial  >> prediction for 15688049142135 is nuc (k=23) | cyto_nucl: 8,  nucl: 6, cyto: 6,  mito: 1, extr: 1 |  | Nucleus: 97% |
| EbMADS11 | 73.9 %: nuclear  26.1 %: mitochondrial  >> prediction for 15688049622134 is nuc (k=23) | nucl: 14 | Nucleus | Nucleus: 92% |
| EbMADS12 | 43.5 %: nuclear  39.1 %: mitochondrial  13.0 %: cytoplasmic  4.3 %: peroxisomal  >> prediction for 15688050262136 is nuc (k=23) | cyto_nucl: 7,  nucl: 6, cyto: 4,  chlo: 2, mito: 2 | Nucleus | Nucleus: 90% |
| EbMADS13 | 73.9 %: nuclear  13.0 %: peroxisomal  8.7 %: cytoplasmic  4.3 %: mitochondrial  >> prediction for 15688050602135 is nuc (k=23) | chlo: 10, nucl: 3, golg_plas: 1 | Nucleus | Nucleus: 87% |
| EbMADS14 | 82.6 %: nuclear  13.0 %: mitochondrial  4.3 %: cytoplasmic  > prediction for 15688050832135 is nuc (k=23) | nucl: 14 | Nucleus | Nucleus: 89% |
| EbMADS15 | 69.6 %: nuclear  30.4 %: mitochondrial  >> prediction for 15688051412134 is nuc (k=23 | nucl: 14 | Nucleus | Nucleus: 87% |
| EbMADS16 | 87.0 %: nuclear  13.0 %: mitochondrial  > prediction for 15688051792134 is nuc (k=23) | cyto: 11, nucl:  2, plas: 1 | Nucleus | Nucleus: 84% |
| EbMADS17 | 82.6 %: nuclear  13.0 %: mitochondrial  4.3 %: cytoplasmic  >> prediction for 15688052052134 is nuc (k=23) | nucl: 14 | Nucleus | Nucleus: 89% |
| EbMADS18 | 43.6 %: nuclear  30.4 %: cytoplasmic  21.7 %: mitochondrial  4.3 %: vesicles of secretory system  >> prediction for 15688052352135 is nuc (k=23) | cyto_nucl: 8,  cyto: 8, nucl: 4,  chlo: 1, plas: 1 | Nucleus | Nucleus: 81% |
| EbMADS19 | 69.6 %: nuclear  13.0 %: mitochondrial  8.7 %: cytoplasmic  8.7 %: peroxisomal  >> prediction for 15688052862135 is nuc (k=23 | nucl: 9, chlo: 2,  mito: 2, plas: 1 | Nucleus | Nucleus: 84% |
| EbMADS20 | 47.8 %: nuclear  26.1 %: mitochondrial  8.7 %: cytoplasmic  4.3 %: plasma membrane  4.3 %: Golgi  4.3 %: peroxisomal  4.3 %: extracellular, including cell wall  > prediction for 15688053212134 is nuc (k=23) | golg: 4, nucl: 3,  vacu: 2, E.R.: 2,  chlo: 1, cyto: 1,  plas: 1 | Nucleus | Nucleus: 89% |
| EbMADS21 | 47.8 %: nuclear  26.1 %: mitochondrial  8.7 %: cytoplasmic  4.3 %: Golgi  4.3 %: plasma membrane  4.3 %: extracellular, including cell wall  4.3 %: peroxisomal  >> prediction for 15688054422134 is nuc (k=23) | golg: 4, nucl: 3,  vacu: 2, E.R.: 2,  chlo: 1, cyto: 1,  plas: 1 | Nucleus | Nucleus: 89% |
| EbMADS22 | 69.6 %: nuclear  13.0 %: cytoplasmic  13.0 %: mitochondrial  4.3 %: vacuolar  >> prediction for 15688055052134 is nuc (k=23) | nucl: 7, cyto: 4,  plas: 1, extr: 1,  cysk: 1 | Nucleus | Nucleus: 86% |
| EbMADS23 | 60.9 %: nuclear  34.8 %: mitochondrial  4.3 %: peroxisomal  >> prediction for 15688055452134 is nuc (k=23) | chlo: 6, cyto: 5,  nucl: 3 | Nucleus | Nucleus: 93% |
| EbMADS24 | 87.0 %: nuclear  8.7 %: mitochondrial  4.3 %: vesicles of secretory system  >> prediction for 15688056052134 is nuc (k=23) | cyto_nucl: 7,  cyto: 6.5, nucl: 4.5,  chlo: 1, mito: 1,  extr: 1 |  | Nucleus: 97% |
| EbMADS25 | 73.9 %: nuclear  8.7 %: vesicles of secretory system  8.7 %: mitochondrial  4.3 %: plasma membrane  4.3 %: cytoplasmic  >> prediction for 15688056362135 is nuc (k=23) | E.R.: 4, nucl: 3.5, cysk_nucl: 2.5,  chlo: 2, mito: 2,  plas: 1, golg: 1 | Nucleus | Nucleus: 89% |
| EbMADS26 | 78.3 %: nuclear  17.4 %: mitochondrial  4.3 %: vesicles of secretory system  >> prediction for 15688056652134 is nuc (k=23) | chlo: 4, plas: 3,  extr: 3, cyto_nucl: 2, nucl: 1.5, cyto: 1.5, vacu: 1 |  | Nucleus: 96% |
| EbMADS27 | 91.3 %: nuclear  8.7 %: mitochondrial  >> prediction for 15688056912134 is nuc (k=23) | nucl: 14 | Nucleus | Nucleus: 90% |
| EbMADS28 | 82.6 %: nuclear  17.4 %: mitochondrial  >> prediction for 15688057142134 is nuc (k=23) | nucl: 11, mito: 1,  plas: 1, cysk: 1 | Nucleus | Nucleus: 87% |
| EbMADS29 | 69.6 %: nuclear  26.1 %: mitochondrial  4.3 %: cytoplasmic  >> prediction for 15688057382136 is nuc (k=23) | chlo: 6, nucl: 5.5, cyto_nucl: 5.16667, nucl_plas: 4.5 | Nucleus | Nucleus: 84% |
| EbMADS30 | 56.5 %: nuclear  39.1 %: mitochondrial  4.3 %: cytoplasmic  >> prediction for 15688057592134 is nuc (k=23) | mito: 8, cyto_nucl: 4.5, cyto: 3, plas: 1 | Nucleus | Nucleus: 84% |
| EbMADS31 | 39.1 %: nuclear  34.8 %: mitochondrial  8.7 %: cytoplasmic  4.3 %: extracellular, including cell wall  4.3 %: plasma membrane  4.3 %: peroxisomal  4.3 %: Golgi  >> prediction for 15688057842136 is nuc (k=23) | nucl: 12, extr: 2 | Nucleus | Nucleus: 89% |
| EbMADS32 | 78.3 %: nuclear  8.7 %: cytoplasmic  8.7 %: mitochondrial  4.3 %: vacuolar  >> prediction for 15688058072133 is nuc (k=23) | nucl: 14 | Nucleus | Nucleus: 93% |
| EbMADS33 | 100.0 %: nuclear  >> prediction for 15688061132135 is nuc (k=23) | nucl: 14 | Nucleus | Nucleus: 89% |
| EbMADS34 | 39.1 %: nuclear  39.1 %: cytoplasmic  21.7 %: mitochondrial  >> prediction for 15688061442135 is nuc (k=23) | nucl: 4, cyto: 4,  plas: 3, pero: 2,  extr: 1 |  | Nucleus: 99% |
| EbMADS35 | 60.9 %: nuclear  30.4 %: mitochondrial  8.7 %: cytoplasmic  >> prediction for 15688061722134 is nuc (k=23) | nucl: 6, cyto: 6,  plas: 2 | Nucleus | Nucleus: 87% |
| EbMADS36 | 73.9 %: nuclear  17.4 %: mitochondrial  8.7 %: cytoplasmic  >> prediction for 15688062082135 is nuc (k=23) | cyto: 7, nucl: 5,  mito: 1, plas: 1 | Nucleus | Nucleus: 89% |
| EbMADS37 | 91.3 %: nuclear  8.7 %: mitochondrial  >> prediction for 15688062392133 is nuc (k=23) | nucl: 14 | Nucleus | Nucleus: 92% |
| EbMADS38 | 82.6 %: nuclear  17.4 %: mitochondrial  >> prediction for 15688063392136 is nuc (k=23) | cyto: 10, nucl: 2,  mito: 1, pero: 1 | Nucleus | Nucleus: 91% |
| EbMADS39 | 82.6 %: nuclear  17.4 %: mitochondrial  >> prediction for 15688064172133 is nuc (k=23) | nucl:10.5,  cyto_nucl: 6,  mito: 1, plas: 1,  cysk: 1 | Nucleus | Nucleus: 84% |
| EbMADS40 | 43.5 %: nuclear  34.8 %: mitochondrial  4.3 %: extracellular, including cell wall  4.3 %: plasma membrane  4.3 %: cytoplasmic  4.3 %: Golgi  4.3 %: peroxisomal  >> prediction for 15688064492134 is nuc (k=23) | chlo: 4, golg: 3,  nucl: 2, extr: 2,  mito: 1, plas: 1,  pero: 1 | Nucleus | Nucleus: 88% |
| EbMADS41 | 39.1 %: nuclear  34.8 %: mitochondrial  8.7 %: cytoplasmic  4.3 %: peroxisomal 4.3 %: Golgi  4.3 %: plasma membrane  4.3 %: extracellular, including cell wall  >> prediction for 15688064802133 is nuc (k=23) | nucl: 12, extr: 2 | Nucleus | Nucleus: 89% |
| EbMADS42 | 91.3 %: nuclear  8.7 %: mitochondrial  >> prediction for 15688065162133 is nuc (k=23) | nucl: 14 | Nucleus | Nucleus: 93% |
| EbMADS43 | 65.2 %: nuclear  17.4 %: mitochondrial  8.7 %: cytoplasmic  8.7 %: peroxisomal  >> prediction for 15688065372135 is nuc (k=23) | cyto: 5.5,  cyto_nucl: 5,  nucl: 3.5, chlo: 2,  extr: 1, pero: 1,  golg: 1 | Nucleus | Nucleus: 86% |
| EbMADS44 | 73.9 %: nuclear  8.7 %: mitochondrial  8.7 %: cytoplasmic  4.3 %: peroxisomal  4.3 %: cytoskeletal  >> prediction for 15688065642135 is nuc (k=23) | nucl: 9, cyto: 3,  chlo: 1, extr: 1 | Golgi apparatus | Nucleus: 87% |
